# GC-Recomposition-Olfactometry (GC-R) and multivariate study of three terpenoid compounds in the aroma profile of Angostura bitters

**DOI:** 10.1038/s41598-019-44064-y

**Published:** 2019-05-21

**Authors:** Arielle J. Johnson, Anna K. Hjelmeland, Hildegarde Heymann, Susan E. Ebeler

**Affiliations:** Department of Viticulture and Enology, University of California, Davis, CA USA

**Keywords:** Small molecules, Analytical chemistry, Olfactory system

## Abstract

Foods and beverage aroma results from multicomponent mixtures of volatile compounds present in the food that interact with olfactory receptors and produce a perceptual response in the brain. However, the perceptual interactions that occur when complex odor mixtures are combined are not well understood. Here we used Gas chromatography-Recomposition-Olfactometry (GC-R) to better understand the role that individual compounds have on the perceived sensory aroma of bitters. Bitters are the concentrated alcoholic extract of flavorful plant materials with a wide range of complex sensory and chemical aroma profiles that have not been extensively studied. Previously, we demonstrated that Angostura bitters are characterized by complex aroma attributes described as *cola*, *ginger*, *orange peel*, and *black pepper* and that the volatile composition of Angostura bitters is predominantly composed of terpenoids. Using GC-R to create in-instrument mixtures of the Angostura headspace extracts, the sensory attributes of Angostura extracts with linalool, α-terpinyl-acetate and caryophyllene omitted were evaluated. The omission experiments demonstrated direct and indirect effects of the individual compounds on the aroma attributes of Angostura bitters, through masking, additive, and synergistic interactions. Caryophyllene in particular, which was present in the headspace extracts at concentration only slightly above sensory threshold levels, had a large and unexpected impact on the sensory properties of the mixtures and may be most responsible for the aromas associated with the whole sample. The GC-R and statistical approaches used here provided valuable tools to reveal relationships among individual compounds and aroma attributes of foods that have not been currently theorized using existing analytical approaches.

## Introduction

Aromas encountered in everyday life are almost always the result of multicomponent mixtures of volatiles. The relationship between the perceived aroma of a mixture of volatiles, its chemical composition, and the aromas of its components is complex and affected by several difficult-to-predict factors. In some cases, the aroma of a mixture is not equivalent to the summation of the aroma qualities of its components^1,2^ and mixtures with as few as three components have been found to have aroma qualities not found in any of these components^3^. It has been estimated that humans are able to discriminate one trillion different odors and that dimensionality of the odor classification space is large (~400 dimensional or larger)^4,5^. However, the way that the brain encodes these complex mixtures is still relatively unknown^6^. Analytical tools that would allow individual components of complex natural odors to be evaluated for their effects on odor quality and variability could provide important information to enhance our knowledge of object perception and coding.

One common method for estimating the relative contribution of volatiles in a sample to the perceived aroma is a calculation called an Odor Activity Value (OAV). OAV represents the measured concentration of a volatile present in a sample divided by its measured sensory detection threshold in a similar matrix^7,8^.

A second method which assesses the volatiles in a sample directly uses GC-Olfactometry (GC-O) with successive dilutions of a sample (*e.g*., Aroma Extract Dilution Analysis (AEDA), CHARM), where a human subject evaluates the aroma and intensity of each peak in a gas chromatogram, and a “dilution factor” required to suppress the detectability of each component is calculated^9–11^.

These separative methods seek to evaluate the potency and quality of a particular volatile at the concentration at which it is found in a particular sample, but, cannot address mixing effects. In many cases, these effects are investigated via reconstitution and omission experiments, typically in a matrix similar to that of the original sample. Having quantified all the volatiles in a sample based on Gas Chromatography-Mass Spectrometry (GC-MS) or GC coupled to another type of detector, a model reconstitution is prepared using chemical standards containing those volatiles (at their in-sample concentrations) hypothesized to contribute to the sample’s aroma, as determined by a certain cutoff OAV or dilution factor. To evaluate the relative contribution of each of these volatiles, several omission mixtures, each of which excludes one compound, are prepared and their aroma is evaluated in comparison to the “whole” reconstitution mixture^11^.

There are several drawbacks to this approach. OAV and dilution factor may not be able to accurately predict whether a compound is above or below its detection threshold, truly “sub-threshold” volatiles may contribute sensory impact when mixed with other volatiles, and instrumental limits of detection may be too high to truly quantify every compound with a sensory impact. “Sub-threshold” compounds, which have been quantified below their putative aroma threshold, are typically excluded, though it has been found that these may play a role in the aroma perception of a mixture^12,13^. Despite containing volatiles calculated to have a sensory impact at their in-sample concentrations, reconstitutions have sometimes been found to differ in aroma from the samples they are supposed to model^14^, reflecting a “reconstitution discrepancy”^15^. While some studies include components calculated to be below their detection threshold in reconstitutions^15^ this is not necessarily a widespread practice. Practically speaking, the time and expense of quantitation, sourcing standards, and reconstitution and omission mixture preparation can be limiting.

In Johnson *et al*.^16^ the technique of and instrumentation required for In-Instrument Gas Chromatography-Recomposition-Olfactometry (GC-R) was developed and used to characterize interactive effects of mixing in the production of lavender aroma. This allowed for the production of reconstitutions in-instrument from volatiles extracted directly from a sample of lavender, with omissions selected by means of a flow switch in real-time during the course of the chromatographic separation. Lavender-like aroma does not have a character impact compound associated with it, and it was found that lavender was sometimes used as a descriptor for the mixtures containing a larger subset of lavender volatiles, but not for smaller and less complex subset mixtures of these volatiles.

Foods and beverages provide the opportunity to study aroma quality of products with cultural and gastronomic interest. Bitters, the highly concentrated alcoholic extractions of flavorful plant materials are particularly interesting due the wide range of flavor profiles available arising from the complex mixtures of ingredients that may be used. Previously, Johnson *et al*.^17^ characterized the flavor-chemical space of commercial aromatic cocktail bitters, and a Partial Least Squares Regression (PLS2) was performed to relate volatile profiling data to sensory profiling data on the bitters used in the experiment. The PLS modeled 60% of the sensory variance in the first two latent variables with 23% of the variance in volatile composition; there were clear associations between volatiles and sensory qualities suggested by the overall similarities in spatial configuration of the two datasets in the PLS, and by proximity of sensory descriptors to individual compounds and groupings of compounds. Some sensory-chemical relationships appeared fairly straightforward, e.g., *clove* aroma was spatially associated with eugenol and its derivatives, which are found in cloves and are often described as “clove-like” (*cinnamon* aroma and cinnamaldehyde was another such familiar or expected relationship). However, the number of compounds (such as terpenoids) present in the dataset without a familiar or straightforward relationship to a single aroma quality left unanswered many questions about how aroma in these mixtures, and or these compounds, is translated from chemistry. In addition, the presence of complex aroma descriptors such as *cola*, *chocolate*, and *ginger*, which aren’t usually associated with a singular impact compound, means that some interactive complexity is probably at play in the aroma of bitters.

In the current work, specific compound-aroma and mixture-aroma relationships of three volatile molecules associated with several aroma descriptors in Angostura bitters were investigated using GC-R reconstitution and omission experiments. This approach allows the additive, masking, and synergistic effects of individual compounds present in mixtures to be comprehensively studied, shedding light on the perceptual properties of complex mixtures.

## Results and Discussion

In a chemical and sensory descriptive analysis of commercial bitters samples, we previously demonstrated that Angostura bitters were positively associated with *cola*, *ginger*, *orange peel*, and *black pepper* aromas (Supplementary Table S1)^17^. More than 50 different volatiles compounds were measured in the headspace of the Angostura bitters, the majority of which were terpenoids with individual aroma characteristics ranging from citrus to floral to herbal (Supplementary Table S2). Using a multivariate statistical approach (Partial Least Squares Regression, PLS1), correlations among the complex aroma attributes and specific compounds of Angostura bitters were obtained (e.g., Supplementary Table S2), although few true causative relationships were observed. This is likely due to mixture dependent interactions that impact overall aroma perception. In the current study three terpenoids (linalool, α-terpinyl acetate, caryophyllene) were selected to further study these mixture dependent effects using in-instrument recombination and omission experiments using the GC-R olfactometry technique previously described^16^. The three terpenoids were chosen based on differences in chemical functional groups, minimal co-elution with other compounds in the GC-MS chromatogram, and some correlations to sensory characteristics (Supplementary Table S1).

**Figure 1:**
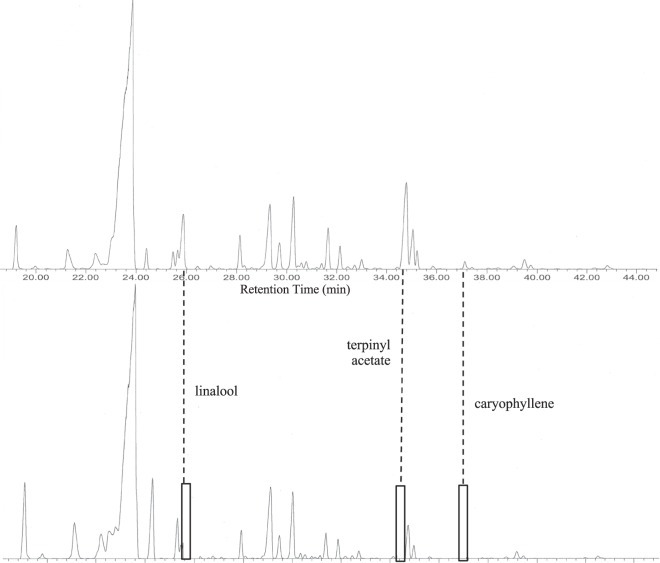
GC-MS chromatograms of (top) Mixture A, the control, uncut sample; and (bottom) Mixture E, with linalool, α-terpinyl acetate, and caryophyllene excluded from the reconstitution.

Using the GC-R technique described by Johnson *et al*.^16^ five different aroma mixtures of Angostura bitters were obtained. Mixture A contained all the components of the headspace sample which were recombined following GC separation and smelled together at the olfactory port (Fig. 1 top). The overall volatile composition of this mixture has been previously reported (Supplementary Table S2)^17^. Mixtures B-D were obtained by excluding the individual peaks for linalool, α-terpinyl acetate, and caryophyllene, respectively and the remaining compounds in the chromatogram were recombined and smelled as a mixture. Finally, for Mixture E all three compounds were excluded and the remaining compounds recombined and smelled at the olfactory port (Fig. 1 bottom). As can be seen in Fig. 1, the peaks for these three compounds were well-resolved from other peaks in the chromatogram and when excluded, no peaks remain in these areas of the chromatogram. All three peaks together account for approximately 5.8% of the total headspace volatile composition of Angostura bitters (calculated as μg/L in relative 2-undecanone equivalents).

For each GC-R mixture, sensory panelists rated the overall aroma intensity from 0–10 and used a check-all-that-apply procedure to provide a description of the sensory attributes. The list of sensory descriptors for the bitters was previously determined^17^ and the number of times each descriptor was applied to each mixture was reported as frequency counts (Table 1). The maximum frequency count of 12 would be obtained if all panelists selected an individual descriptor across each replication,

**Table 1:** Aroma properties of GC-R reconstitution mixtures listed by volatiles excluded and calculated odor activity values (OAV) of excluded compounds.

Mixture	A	B	C	D	E
Volatiles excluded	None	Linalool	Alpha Terpinyl Acetate	Caryophyllene	Linalool, Alpha terpinyl acetate, Caryophyllene
**Linalool**					
Headspace concentration (ug/L, 2-undecanone equivalents)		42	535	84	
Odor Threshold in water (ug/L)		6		64	
Calculated Odor Activity Value (OAV)		7		1.3125	
intensity	5.5	4.4	4.6	5.1	4.8
cola	8	7	4	6	4
ginger	3	4	1	0	3
orange peel	7	6	8	9	6
cardamom	3	1	0	1	4
anise	5	3	4	4	5
clove	8	8	3	10	7
orange candy	6	6	7	5	7
cinnamon	7	3	3	3	4
lime peel	6	6	6	4	2
tea	1	0	0	1	2
vanilla	3	6	3	6	1
nutmeg	5	3	1	3	4
root beer	5	1	2	5	1
dried fruit	1	0	1	0	0
wood	4	6	5	2	5
brown sugar	3	3	4	3	3
molasses	2	1	0	0	0
black pepper	5	2	3	0	2
grapefruit	2	0	2	0	2
caraway	1	0	1	0	0
juniper	3	1	0	0	2
earthy	1	1	2	4	3
alfalfa/hay	0	0	0	0	0
chile	0	0	0	0	1
celery seed	1	0	0	0	1
mint	4	7	5	7	7
chocolate	1	1	1	2	1
soapy	2	4	2	3	1
green	1	2	2	2	3

“Intensity” is the average overall aroma intensity rated by three panelists from 1–10 for each mixture. Aroma descriptors expressed as overall counts for each descriptor for each sample across three panelists and four replicates. Odor thresholds reported by Guadagni *et al*.^8^.

The overall average aroma intensity was similar for all aroma mixtures, ranging from 5.5 (Mixture A with no compounds removed) to 4.4 (Mixture B with only linalool removed) (Table 1). There are clear differences in the average frequency of aroma descriptors used for each of the mixtures (Table 1). For example, the *cola* and *clove* descriptors were selected most frequently (selected 8 of 12 times) for Mixture A, which contained all the headspace compounds of the Angostura bitters sample. The highest average frequency (selected 10 of 12 times) was obtained for the *clove* descriptor with Mixture D.

**Figure 2:**
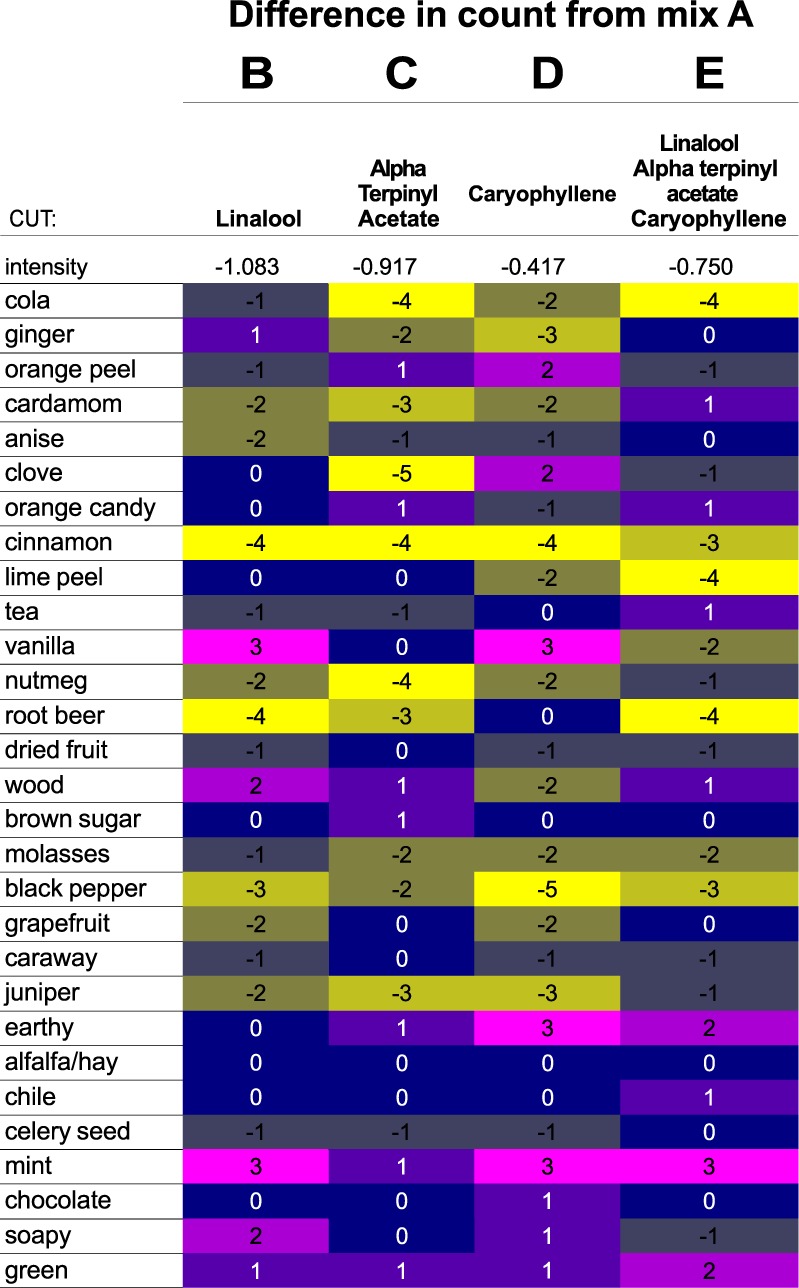
Differences in aroma qualities of mixtures with volatiles excluded compared to the control mixture. Decreases in descriptor count for experimental conditions highlighted in yellow, increases in descriptor count highlighted in magenta.

In order to better compare and visualize differences among the mixtures, the overall intensity and frequency counts for each omission mixture were compared to the control, Mixture A (Fig. 2). For each descriptor in each experimental sample, the number of frequency counts relative to the number of frequency counts for the control sample (Mixture A, nothing cut, every peak cryotrapped and combined) reflects the sensory role that individual compound plays in the aroma of the control mixture. For example, no change in frequency counts for a descriptor when a compound is omitted shows that that compound does not impact that aroma (*e.g*., linalool removal does not affect *clove* aroma of the recomposition mixture; Fig. 2). A decrease in frequency count upon the omission of a compound shows that that compound contributes to that aroma, with larger decreases indicating a greater effect (*e.g., cola* aroma decreases upon removal of linalool and α-terpinyl acetate from the mixture, with a greater impact resulting from removal of α-terpinyl acetate). An increase in frequency counts for an attribute, upon removing a compound, indicates that compound masks or otherwise dampens or modulates the perception of that attribute (*e.g*., *ginger* aroma increases upon removal of linalool from the recomposition mixture). In Fig. 2 these differences are highlighted as a heatmap where increases in frequency counts for a particular descriptor shift proportionally from blue to purple/magenta, and decreases in frequency counts for a given descriptor are shift proportionally from green to yellow.

For each of the individual compounds we can compare how the aroma attributes change when the compound is removed from the mixture (Fig. 2). Linalool contributes most to the aromas of *cinnamon*, *root beer*, and *black pepper* in the reconstitution mixtures. This is reflected by a decrease in frequency of use of these descriptors (−3 or −4 counts relative to the control) for each of these descriptors when linalool is excluded from the mixture. Linalool makes a small contribution to *cardamom*, *anise*, *nutmeg*, *grapefruit*, and *juniper* aroma qualities in the mixture (decrease of -2 frequency counts relative to the control). Finally, the *vanilla*, *mint*, *wood*, and *soapy* aroma attributes are used more frequently (+2 to +3 counts compared to the control) when linalool is cut from the mixture, indicating that linalool masks these attributes.

α-Terpinyl acetate contributes most to the aromas of *cola*, *cardamom*, *clove*, *cinnamon*, *nutmeg*, *root beer*, and *juniper*, with small contributions to *ginger*, *molasses*, and *black pepper* aromas in the reconstitution mixtures (Fig. 2). The frequency of use of each of these sensory attributes decreases in Mixture C compared to the control. α-Terpinyl acetate does not appear to contribute any significant masking effects since no aroma attributes increase in frequency of use by 2 or more counts relative to the control when this compound is removed.

Caryophyllene contributes most to aroma qualities of *ginger*, *cinnamon*, *black pepper*, and *juniper* in the reconstitution mixtures (decrease in frequency counts of −3 to −5 relative to control Mixture A), with smaller contributions to *cola*, *cardamom*, *lime peel*, *nutmeg*, *wood*, and *grapefruit* aromas (decrease in frequency counts of −2 relative to control). Caryophyllene masks aromas of *vanilla*, *earthy*, *mint*, *orange peel*, and *clove* (increase in frequency counts of +2 or +3, relative to control).

Frequency count differences between Mixture E, which omitted all three test compounds, and the control mixture reveal mixing-dependent perceptual effects, where the contribution of one compound to a descriptor is affected by the contributions of the other two, or where omitting all three compounds has an effect that could not be predicted from examining the individual effects of cutting single compounds. In Mixture E, *cola*, *clove*, *cinnamon*, *root beer*, *molasses*, *mint* and *black pepper* aroma qualities all appear to show surprisingly similar changes when all three compounds are simultaneously cut as when compared to the aroma qualities in aggregate for mixtures B, C, and D, when only one of the compounds is cut. For each of these descriptors, the change in frequency counts for mixture E compared to the control either mirror the sample (B, C, or D) with the largest change in frequency counts, or reflect an averaging where cutting one compound increased counts and cutting another decreased them.

*Lime peel* aroma appears to have some synergistic effects associated with it, as it has no change in frequency counts after cutting either linalool or α-terpinyl acetate, and only a small decrease in counts when caryophyllene is cut; however, there is a marked decrease in frequency counts when all three compounds are cut. This suggests that while none of the three compounds has a strong *lime peel* quality on their own, this aroma characteristic depends in part on the impact of having all three compounds present together in the mixture.

For other aroma qualities—*ginger*, *cardamom*, and *vanilla*— cutting all three compounds in Mixture E leads to an opposite effect compared to the mixtures where only a single compound is cut. For the aroma attribute *ginger*, cutting either α-terpinyl acetate or caryophyllene leads to a decrease in frequency counts, but cutting all three compounds from the reconstitution mixture leads to no change in the number of counts for experimental Mixture E compared to the control Mixture A. The same is true for *cardamom*, for which all three compounds appear to contribute in isolation (cutting any one of them leads to a small decrease in counts), but omitting all three compounds at once leads to an essentially unchanged descriptor count compared to the control. In the case of *vanilla*, omitting either caryophyllene or linalool increases the descriptor count, but removal of all three compounds slightly decreases the descriptor count; it is unclear why this would be the case for this odor quality in particular.

**Figure 3:**
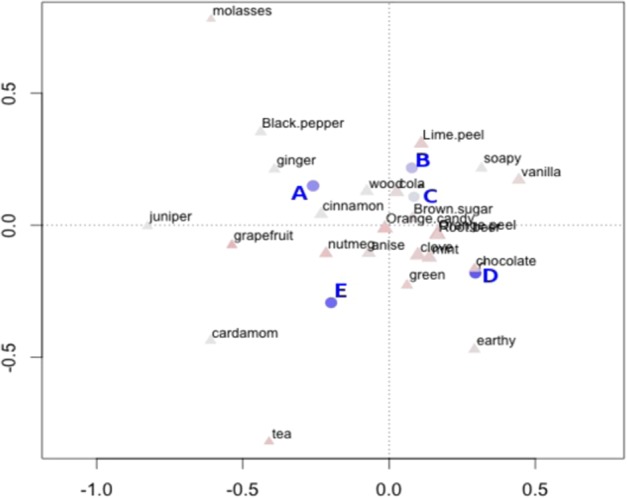
Correspondence analysis biplot of mixtures (blue circles, blue text) and descriptor counts (red triangles, black text). Dimension 1 (x) Variance explained = 37.2%, Dimension 2 (y) variance explained = 27.6%. Mixture A: control, uncut sample; Sample B: linalool omitted; Mixture C: α-terpinyl acetate omitted; Mixture D: caryophyllene omitted; Mixture E: linalool, α-terpinyl acetate and caryophyllene omitted.

To further evaluate the similarities and differences in the mixtures, a correspondence analysis was performed taking into account all of the sensory descriptors. Figure 3 shows a biplot of the correspondence analysis performed on the frequency data summed over all panelists and replicates. In this biplot, mixtures are represented as circles and descriptors as triangles. Spatially, the correspondence analysis biplot suggests that in a multivariate context, mixtures B and C are most similar to each other, and each are fairly different from mixtures A, D, and E. This suggests that omitting linalool (B) and α-terpinyl acetate (C) has similar effects overall on the aroma profile, while omitting caryophyllene (D) produces a different effect. There was also good agreement among the three panelists about the relative similarities and differences of the mixtures as demonstrated by Multiple Factor Analysis (Supplementary Fig. S1).

In the correspondence analysis, positional proximity of a mixture to descriptors reflects correlations between those descriptors and that sample mixture (Fig. 3). Since the samples evaluated here have omitted specific compounds from the recomposition mixture, the descriptors that are negatively correlated (i.e., in opposite quadrants) to a sample are the ones to which the compound that has been cut in that sample are correlated. For example, mixture D, which omits caryophyllene, plots closely to *chocolate*, *green*, and *earthy* descriptors, which suggests that caryophyllene is negatively correlated to those aromas; caryophyllene itself would plot at a point directly reflected through the origin, close to *ginger*, *black pepper*, *wood*, and *cinnamon* aroma qualities. This is also reflected in Fig. 2, where omitting caryophyllene (as discussed above) leads to a slight increase in the number of frequency counts for *green* and *chocolate*, a slightly larger increase in frequency counts for *earthy* aroma, and a decrease in the number of counts for *cinnamon*, *black pepper*, *ginger*, *juniper*, and *wood* aromas.

Importantly these results indicate that omitting all three compounds either unmasks or reduces aromas differently than omitting any single compound. Aroma descriptors such as *cola, soapy*, *vanilla*, *lime peel*, *brown sugar*, *orange peel*, and *orange candy* appear to be the most dependent on more than one compound, as they plot directly opposite mixture E, which excludes all three compounds under investigation. The position of mixture D, which omits caryophyllene, puts the position of caryophyllene at a point reflected directly through the origin of the plot—essentially, very close to mixture A, the uncut control sample. This suggests that, of the three compounds studied, caryophyllene is the most directly responsible for the aromas associated with the whole sample. In future work, inclusion of more panelists as well as a ‘negative control’ where the aroma of a mixture is monitored before and after removal of a compound that has no effect on perception, would help to further characterize the overall variability of the data.

GC-R was developed with a view to simplifying the elucidation of the role of both individual volatiles to complex aromas, and interactions between volatiles. And application of GC-R to the Angostura volatiles mixtures did allow for rapid, causative determinations of the relationships between a particular compound and particular odor qualities, which could be examined only correlatively in the previous study of bitters aroma. Interestingly, however, the results of the current work show that even in experiments focusing on the roles of a limited number of compounds, the sensory results of investigating each are still quite complex. Differential contributions of compounds to the intensity of some aromas and not others, as well as evidence for masking of other sensory qualities once specific compounds are removed from the reconstitution, are evident. Negative spatial correlation in the correspondence analysis between the sample (E) with multiple compounds removed and a number of aromas that do not have an impact compound (such as *cola*, *root beer*, and *soapy*) reflects an association between the lack of these compounds and the lack of those aromas, or more simply, an association between all three compounds together and those aromas. That these compounds appear to enhance some aromas and suppress others suggests a complex relationship to perception, mediated through mixing, which has also been described elsewhere^1,12,18–21^.

Both linalool and caryophyllene have fairly low putative OAVs in this sample, 7 in the case of linalool and 1.3 in the case of caryophyllene (Table 1). While caryophyllene in a traditional reconstitution and omission study would be considered to be barely detectible, removing it by GC-R tends to have a larger and unpredicted effect on aroma quality than removing either linalool or α-terpinyl acetate. Again, it is important to emphasize that using GC-R, these sensory effects could be analyzed directly from a headspace extract of the sample of Angostura bitters, without a quantitation step, without determining odor thresholds, and without performing time-intensive aroma extract dilution analysis.

The simplicity of performing GC-R could be used to investigate more compound-level sensory interaction effects in complex samples, and allows for greater detail about differences in multiple descriptors than a simple forced choice difference test reveals. This suggests that further development and application of methods, statistical, instrumental, or otherwise, to elucidate interactive and synergistic sources for such aromas in these and other samples (some of which may already be well-characterized by current methods) may reveal relationships that have not been currently theorized using existing analytical approaches.

## Materials and Methods

### Bitters

Angostura bitters were purchased commercially. These were the same samples as used in Johnson *et al*.^17^.

### Sample preparation and extraction

A 2.5 mL sample of bitters was diluted with 7.5 mL of deionized water in a 20 mL amber glass headspace vial and sealed with a crimp cap having a PTFE-faced silicone lining (Supelco, St. Louis, MO). The sample was shaken at 500 rpm for one minute, after which a Solid Phase Microextraction fiber (2 cm length, 50/30 μm divinylbenzene/carboxen/polydimethylsiloxane coating, Supelco, Bellefonte, PA) was immediately used for extraction. The fiber was exposed to the headspace of the vial for 30 minutes at room temperature, then withdrawn and immediately desorbed in the GC-R inlet.

### Instrument and analysis conditions

An Agilent model 6890 gas chromatograph/5972 mass spectrometer detector (GC-MSD) modified with Deans Switch, auxiliary pressure controller, cryotrap, and olfactometry port as described in Johnson *et al*.^16^ was used, with the modification of an Agilent three-way splitter (instead of a Gerstel four-way splitter) splitting the eluent of volatiles between the mass spectrometer and the olfactory port. Separation was performed using a 30 m × 50 mm i.d. × 0.32 μm film thickness DB-5MS column (J&W, Folsom, CA). The inlet was maintained at 270 °C in splitless mode. Helium was used as the carrier gas and was held at a constant pressure of 12.6 psi. The auxiliary pressure controller was maintained at 3.4 psi. Following sample extraction, the SPME assembly was introduced manually into the GC inlet and allowed to desorb for a total of 10 minutes. The oven was held at 60 °C for 3 minutes, then ramped to 150 °C at a rate of 3 °C/min, then ramped to 325 at a rate of 30 °C/min and held for 5 min for a total runtime of 49 minutes. Both the olfactory port transfer line and the MSD transfer line were maintained at 300 °C. After a 4 minute solvent delay, the mass spectrometer scanned from *m/z* 50–300. The eluent from the GC column, minus any retention time segments cut by the Deans Switch, were collected in a cryotrap (Micro Cryo- trap and model 971 controller, Scientific Instrument Services, Ringoes, NJ) using liquid carbon dioxide. The switch was programmed in the “runtime” tab of the Enhanced Chemstation Software (Hewlett Packard, version B.01.00) to direct the flow over the course of the runtime as desired by the operator.

### Sensory analysis

Three volatile compounds—linalool, α-terpinyl acetate, and *trans*-caryophyllene— were chosen based on criteria of functional group and chemical class diversity, minimal co-elution with other compounds in the GC-MS chromatogram, and correlation to sensory characteristics (see Materials and Methods and Supplementary Table S1) to be the basis of recomposition mixtures for sensory evaluation. Literature orthonasal detection thresholds in water for caryophyllene and linalool^8^ were used to calculate putative odor activity values, based on relative headspace concentrations determined previously^17^. Five recomposition samples were produced (see Table 1) by cutting sections of the chromatogram in real time to waste based on retention time. Mixture A incorporated all volatiles with no cuts; Mixture B incorporated all headspace volatiles in Angostura bitters except linalool; Mixture C incorporated all volatiles except α-terpinyl acetate; Mixture D incorporated all volatiles except caryophyllene; and Mixture E incorporated all volatiles except linalool, α-terpinyl acetate, and caryophyllene. Three panelists (UC Davis students, 2 female, ages 26–33) smelled each recomposition mixture at the olfactory port, rated the overall aroma intensity of the sample from 1–10, and indicated all descriptors that applied to the sample from a list of aroma descriptors previously generated by the sensory panel and linked to reference standards^17^. Panelists were blind to the sample identity and four replicates of each recomposition mixture were performed per panelist. Use of human subjects for this study was reviewed by the University of California, Davis Institutional Review Board and was granted exempt status (Category 6).

### Statistical analysis

Preliminary identification of candidate compounds for omission was performed via PLS1 analysis (Supplementary Table S1) of the descriptive analysis data and volatile profiling data; the whole volatile dataset was used as the x-variable and each aroma descriptor was a y-variable in Unscrambler (CAMO Software AS, Oslo, Norway)^17^.

Using the data generated by the three panelists in the current study, the mean aroma intensity for each mixture was calculated, and the total number of times each attribute was identified for each mixture was calculated and reported as frequency counts. Correspondence analysis (CA) on the aggregated, check-all-that-apply datasets and Multiple Factor Analysis (MFA) comparing each panelists’ datasets were performed using the “ca” and “FactoMineR” packages for the R statistical program (R Foundation for Statistical Computing, Vienna, Austria), respectively.

## Supplementary information

Supplementary Information

## Data Availability

The data generated or analyzed during this study are included in this published article and its Supplementary Information files as well as in Johnson *et al*.^17^ Data is also available from the corresponding author on reasonable request.
