# Supplementary material for: GC-Recomposition-Olfactometry (GC-R) and multivariate study of three terpenoid compounds in the aroma profile of Angostura bitters
Source: Sci Rep. 2019 May 21;9:7633. doi: 10.1038/s41598-019-44064-y (PMC6529406; doi:10.1038/s41598-019-44064-y)
Supplement: Supplementary file 1 — Supplementary Information [file 41598_2019_44064_MOESM1_ESM.pdf]

Supplementary Information

GC-Recomposition-Olfactometry (GC-R) and multivariate study of three terpenoid compounds in the aroma profile of Angostura bitters

Arielle J. Johnson, Anna K. Hjelmeland, Hildegarde Heymann, Susan E. Ebeler

Supplementary Table S1. PLS1 analysis positions in biplot of compounds and Angostura sample compared to specific descriptors (positions of descriptors is approximately 0.2 along each PC), from bitters descriptive analysis data (Johnson et al.[17]).

	Angostura	linalool	Alpha terpinyl acetate	caryophyllene
		cola		
PC1	6.787	0.192	1.56E-02	0.176
PC2	-1.279	-3.33E-02	-0.109	-0.139
		ginger		
PC1	4.486	0.165	0.139	0.137
PC2	-1.583	-3.02E-02	-6.22E-02	-0.153
		orange peel		
PC1	4.027	0.142	0.115	0.112
PC2	-2.171	-3.51E-03	-0.138	-0.148
		clove		
PC1	-0.718	0.118	-5.11E-02	6.41E-02
PC2	2.818	-0.106	0.11	-0.108
		root beer		
PC1	0.305	1.26E-02	-6.45E-02	-1.78E-02
PC2	2.969	7.74E-02	0.226	8.00E-02
		black pepper		
PC1	6.698	-0.103	5.57E-02	-1.69E-02
PC2	-5.247	5.68E-02	6.79E-02	0.112
		earthy		
PC1	-4.144	-0.173	-0.114	-0.123
PC2	1.234	3.31E-02	0.123	0.176
		mint		
PC1	-2.596	-6.94E-02	-0.103	-6.99E-02
PC2	3.906	9.98E-02	0.171	0.14

Supplementary Table S2. Headspace volatile composition of Angostura bitters (Adapted from Johnson et al.[17]).

Compound Name[1]	Compound Class[2]	Relative Headspace Concentration (ug/L, 2-undecanone equivalents)
limonene	T	4521
beta-phellandrene	T	2123
p-cymene	T	736
gamma-terpinene	T	715
alpha-terpinyl acetate	T	535
ni.h		367
sabinene	T	330
alpha-pinene	T	286
terpinolene	T	275
beta-myrcene	T	205
3-carene	T	137
ni.j		134
ni.d		111
caryophyllene	T	84
Beta-pinene	T	79
eucalyptol	T	71
Alpha-thujene	T	50
alpha,p-dimethylstyrene	B/P	42
linalool	T	42
Beta-eudesmol	T	42
beta-trans-ocimene	T	32
ni.m		27
eugenol	B/P	26
cis-beta-ocimene	T	23
lavandulyl acetate	T	23
beta-cadinene	T	23
geranyl acetate	T	22
myristicin	T	17

decanal	A	16
safrole	B/P	16
nerol acetate	T	15
humulene	T	13
calamene	T	13
camphene	T	11
copaene	T	11
bornyl acetate	T	11
ni.l		10
eudesmol	T	9.4
camphor	T	8.4
ni.n		7.5
p-menth-1-en-8-ol	T	6.8
linalyl acetate	T	6.5
octanal	A	4.4
Alpha-curcumene	T	4.3
ethyl octanoate	E	3.9
Alpha-calacorene	T	3.8
nerolidol	T	2.7
caryophyllene oxide	T	1.8
cinnamaldehyde	B/P	1.4
hexanal	A	0.9
methyleugenol	B/P	0.7
cubenol	T	0.5
Sum		11,257

[1]ni: compound not identified; see Johnson et al. (2015) for details

[2] T: Terpenoid; B/P: Benzenoid/Phenypropanoid; A: Aliphatic aldehyde; E: Aliphatic ester

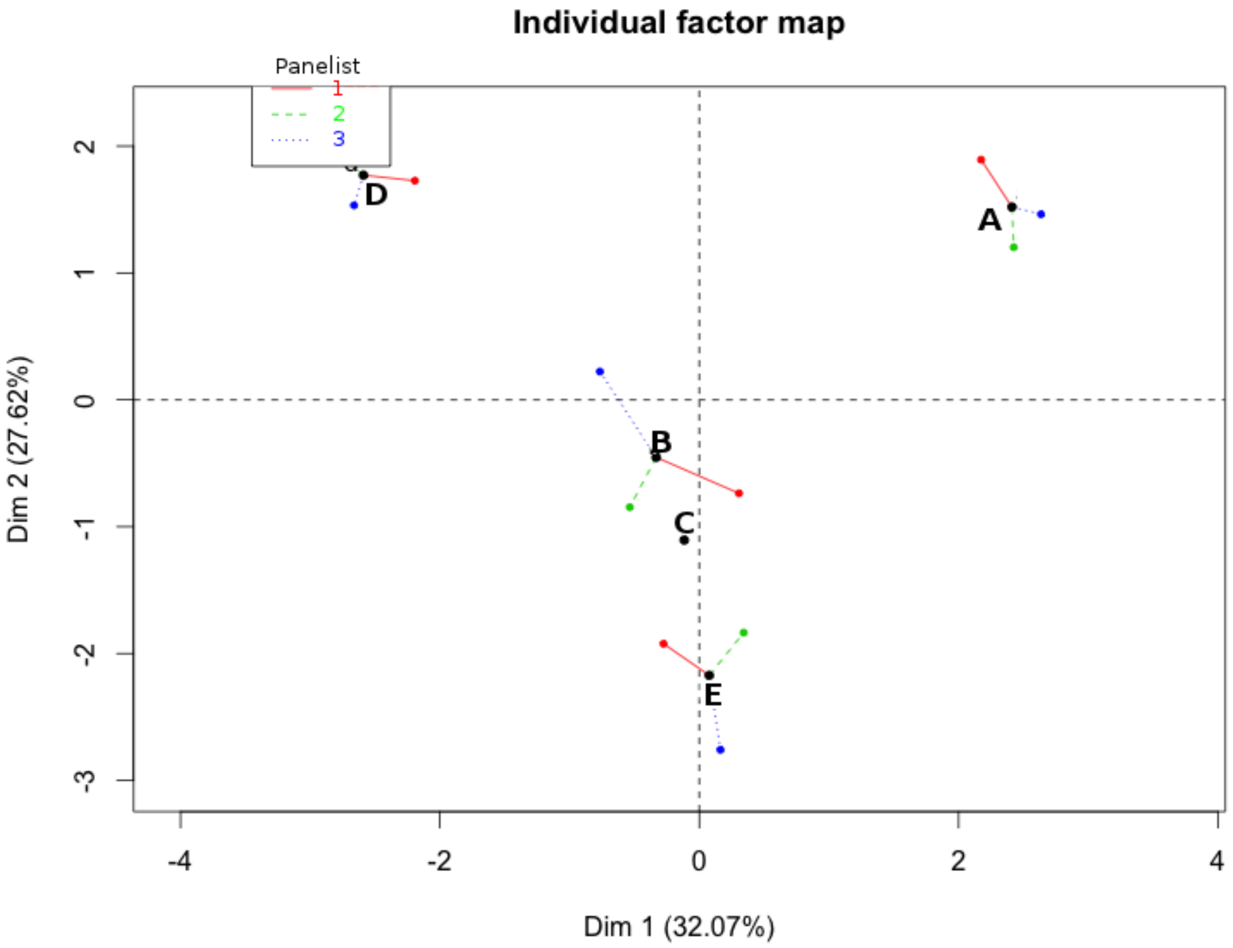

Supplementary Figure S1. Multiple Factor Analysis (MFA) individual factor map comparing panelists' individual map positions with consensus map positions. Disagreement over consensus positions is denoted by colored vectors for each panelist. Generally, there is good agreement about the relative similarities and differences of each mixture as determined by each panelist, with mixtures A and D each in an isolated quadrant and more spatial similarity for mixtures B, C, and E. This plot shows a similar separation of mixtures A, D, and E from each other as was evident from the correspondence analysis, however, mixtures B and C are somewhat closer and therefore more similar to mixture E than denoted by the correspondence analysis. Positions in the Correspondence Analysis are reflective of summing the incidence of 12 possible hits (given three panelists and four replicates) for any given mixture-descriptor pair, while each panelist-derived position in the Multiple Factor Analysis (MFA) consensus map is derived from a maximum of 4 hits. While MFA is a useful check for panelist agreement, the correspondence analysis may be more information-rich as a basis for comparisons among mixtures.
